# Schwann cells are activated by ATP released from neurons in an *in vitro* cellular model of Miller Fisher syndrome

**DOI:** 10.1242/dmm.027870

**Published:** 2017-05-01

**Authors:** Umberto Rodella, Samuele Negro, Michele Scorzeto, Elisanna Bergamin, Kees Jalink, Cesare Montecucco, Nobuhiro Yuki, Michela Rigoni

**Affiliations:** 1 Department of Biomedical Sciences, University of Padua, Padua 35131Italy; 2 Division of Cell Biology, The Netherlands Cancer Institute, Amsterdam 1066 CX, The Netherlands; 3 CNR Institute of Neuroscience, Padua 35131, Italy; 4 Department of Neurology, Mishima Hospital, Niigata 940-2302, Japan

**Keywords:** ATP, Calcium, cAMP, Phospho-CREB, Miller Fisher syndrome, Schwann cells

## Abstract

The neuromuscular junction is exposed to different types of insult, including mechanical trauma, toxins and autoimmune antibodies and, accordingly, has retained through evolution a remarkable ability to regenerate. Regeneration is driven by multiple signals that are exchanged among the cellular components of the junction. These signals are largely unknown. Miller Fisher syndrome is a variant of Guillain–Barré syndrome caused by autoimmune antibodies specific for epitopes of peripheral axon terminals. Using an animal model of Miller Fisher syndrome, we recently reported that a monoclonal anti-polysialoganglioside GQ1b antibody plus complement damages nerve terminals with production of mitochondrial hydrogen peroxide, which activates Schwann cells. Several additional signaling molecules are likely to be involved in the activation of the regeneration program in these cells. Using an *in vitro* cellular model consisting of co-cultured primary neurons and Schwann cells, we found that ATP is released by neurons injured by the anti-GQ1b antibody plus complement. Neuron-derived ATP acts as an alarm messenger for Schwann cells, where it induces the activation of intracellular pathways, including calcium signaling, cAMP and CREB, which, in turn, produce signals that promote nerve regeneration. These results contribute to defining the cross-talk taking place at the neuromuscular junction when it is attacked by anti-gangliosides autoantibodies plus complement, which is crucial for nerve regeneration and is also likely to be important in other peripheral neuropathies.

## Abstract (precis)

**Summary:** ATP released by degenerating neurons participates in neuron-Schwann cell communication in an *in vitro* model of Miller Fisher syndrome and activates Schwann cell pro-regenerative properties.

## INTRODUCTION

The neuromuscular junction (NMJ) is composed of the motor axon terminal (MAT), capped by perisynaptic Schwann cells (PSCs) and parajunctional fibroblasts, and the muscle fiber. A basal lamina separates the pre- and post-synaptic compartments and covers PSCs (Sanes and Lichtman, 1999; Court et al., 2008). This specialized synapse is often exposed to different kinds of pathogen, including neurotoxins, and can be affected by disorders of autoimmune origin, such as the Miller Fisher syndrome (MFS), a subtype of Guillain–Barré syndrome (GBS). Clinical hallmarks of MFS are ophthalmoplegia, ataxia and areflexia. GBS syndrome encompasses several conditions of autoimmune origin with a different distribution of weakness in the limbs or in cranial nerve-innervated muscles, and thus can have highly different prognoses, ranging from spontaneous complete recovery (as for MFS) to a poorer outcome (Yuki and Hartung, 2012).

For its essential role in life and survival, the NMJ has retained throughout vertebrate evolution an intrinsic ability to regenerate, unlike central synapses. A crucial role in NMJ recovery of function is played by Schwann cells (SCs), which undergo an injury-induced reprogramming toward a regenerative phenotype (Jessen and Mirsky, 2016) and release an array of molecules capable of promoting neuronal regeneration. The importance of PSCs in MAT regeneration has been documented in a number of NMJ pathological conditions. In an *ex vivo* model of MFS it has been reported that PSCs processes wrap around MAT debris following degeneration induced by the combination of an anti-GQ1b IgM antibody plus complement (O'Hanlon et al., 2001). To date, however, the current understanding of the role of PSCs in these autoimmune neuropathies is mostly phenomenological, and molecular studies are therefore needed. Several different signals are thought to be generated by all the components of the NMJ, and have been only partially identified.

To better elucidate the molecular and cellular events driving the PSC response to MAT damage in MFS, we have recently set up *in vitro* and *in vivo* models of MFS (Rodella et al., 2016). The combination of a monoclonal IgG antibody against GQ1b/GT1a polysialogangliosides (named FS3, Koga et al., 2005) plus a source of complement is the ‘pathogen’ responsible for the reversible injury of the MAT observed in this autoimmune neuropathy. FS3 binds to presynaptic terminals at the NMJ and to isolated primary neurons, where it activates the complement cascade, with deposition of the membrane attack complex (MAC) on the neuronal surface. A rapid degeneration of nerve terminals occurs, triggered by calcium overload and mitochondrial impairment. We found that hydrogen peroxide (H_2_O_2_), produced by dysfunctional mitochondria, rapidly reaches SCs in co-culture with primary neurons, activating their regenerative program (Rodella et al., 2016). As the MAC complex is very large and assembles very rapidly, it is likely that a large amount of ATP can rapidly efflux from the damaged MAT. Here, we tested this possibility and found that ATP is indeed released, and that it acts as a danger-signaling molecule for SCs. We also investigated the intracellular signaling pathways activated by ATP in SCs.

## RESULTS

### ATP is released by degenerating neurons

**Fig. 1.**
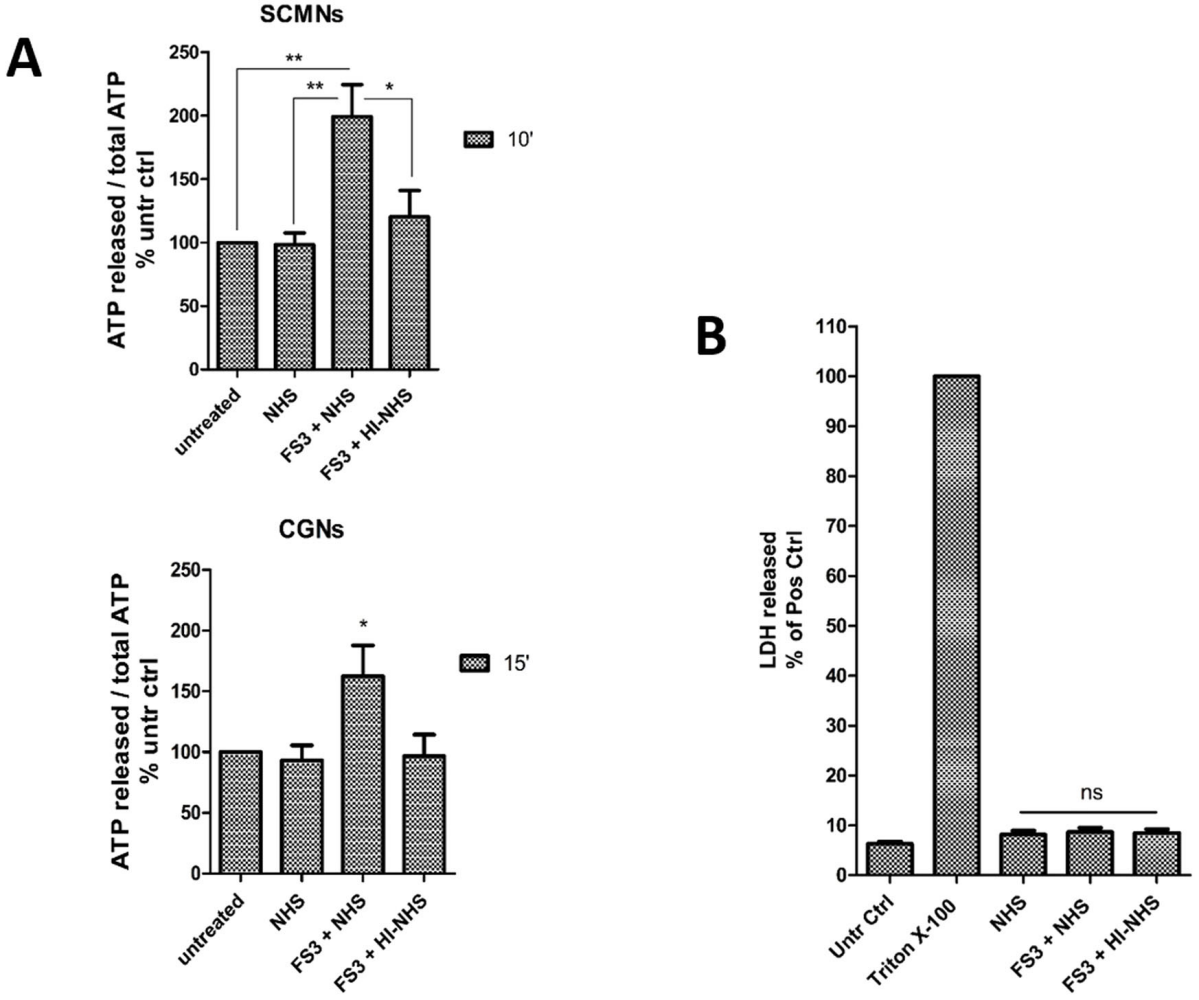
**ATP is released by neurons treated with anti-GQ1b antibody plus complement.** (A) Time-course of ATP release by SCMNs and CGNs exposed to NHS, FS3+NHS or FS3+HI-NHS for 10 min (SCMNs) or 15 min (CGNs). The amount of release is expressed as a percentage of total ATP relative to untreated samples. *P<0.05; **P<0.01; *N*=7 (Student's *t*-test, unpaired, two-tailed). (B) LDH activity measured in the supernatant of CGNs treated as in A, and expressed as a percentage of Triton X-100-treated samples. *N*=5; ns, not significant.

Spinal cord motor neurons (SCMNs) or cerebellar granular neurons (CGNs) exposed to FS3 plus normal human serum (NHS) as a source of complement (FS3+NHS) rapidly release ATP in the supernatant, measured by a luminometric assay, as shown in Fig. 1A. This effect is dependent on both FS3 and complement, as no release is detectable upon exposure to NHS alone or when FS3 is combined with heat-inactivated serum (FS3+HI-NHS). Under the same experimental conditions, no lactate dehydrogenase (LDH) activity was detected in the cell supernatant (Fig. 1B), meaning that ATP is not released as a mere consequence of cell lysis caused by treatment with FS3+NHS.

### Neuronal ATP triggers calcium spikes in Schwann cells

**Fig. 2.**
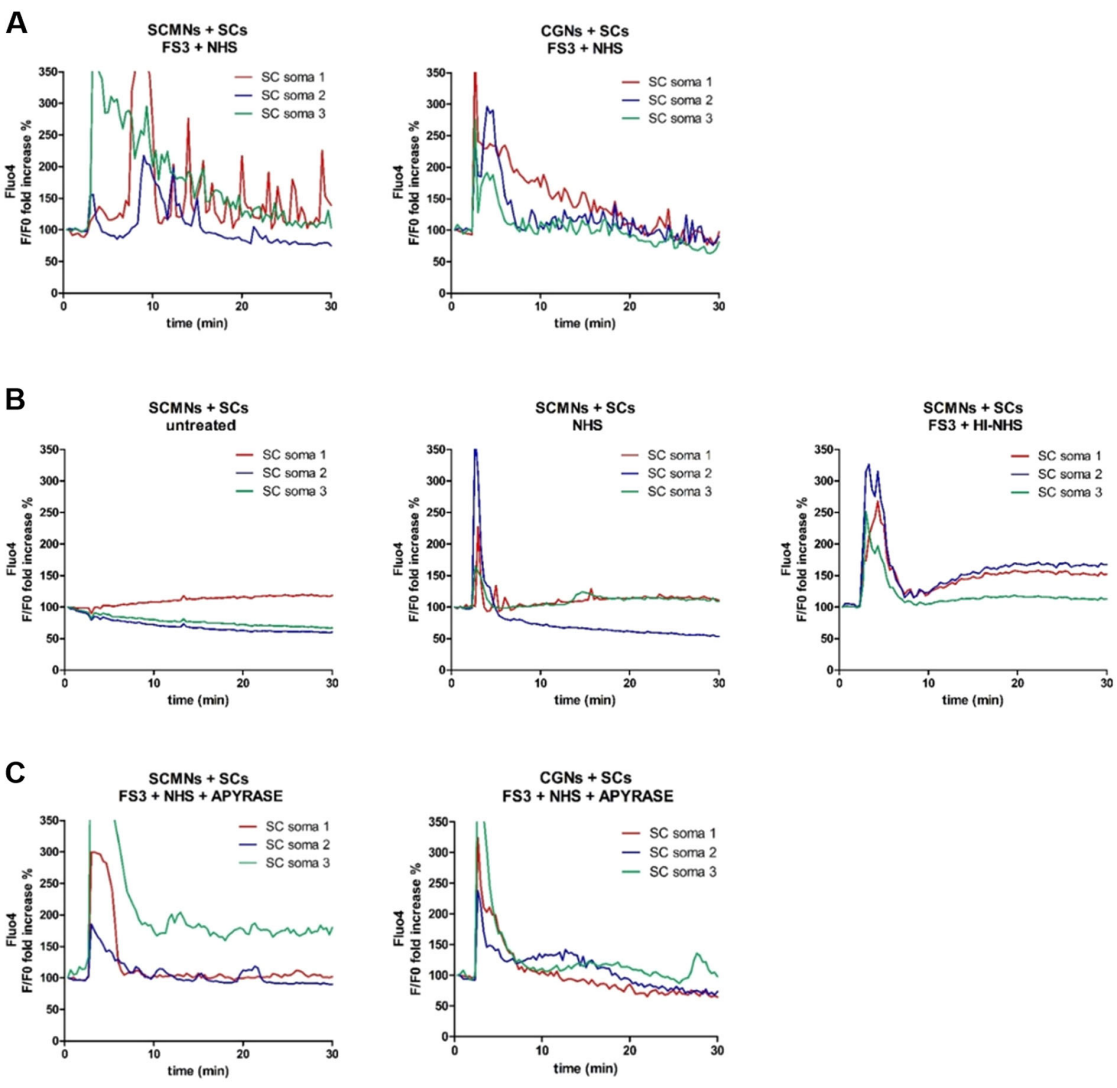
**Neuronal ATP triggers calcium spikes in Schwann cells co-cultured with neurons treated with anti-GQ1b antibody plus complement.** (A,B) Co-cultures of primary SCs and SCMNs or CGNs were loaded with Fluo4 AM and exposed to FS3+NHS, saline, NHS or FS3+HI-NHS for 30 min. Intracellular calcium changes are quantified. (A) In co-cultures exposed to FS3+NHS, calcium spikes are observed in SCs. (B) No calcium increase is detectable in SCs in co-cultures with SCMNs upon incubation with saline, NHS or FS3+HI-NHS. (C) Apyrase preincubation nearly abolishes calcium spikes in SCs upon FS3+NHS treatment. Representative traces are reported; *N*=3.

We next wondered whether SCs might sense and respond to neuronal ATP, given that terminal SCs express on their surface different types of purinergic receptors. We found recently that primary SCs respond to exogenous ATP with cytosolic calcium concentration ([Ca^2+^]) spikes (Negro et al., 2016). When SCMNs or CGNs in co-culture with primary SCs are exposed to FS3+NHS, bulges or varicosities rapidly form along neurites, leaving SCs unaffected (Rodella et al., 2016). Intracellular [Ca^2+^] levels progressively rise within these characteristic rounded structures and in neurites (Rodella et al., 2016), and, immediately after, [Ca^2+^] spikes are observed in SCs (Fig. 2A and Movies 1 and 3). No changes in [Ca^2+^] occur under control conditions and upon exposure to NHS or to FS3+HI-NHS (Fig. 2B). A cytosolic [Ca^2+^] spike in SCs is observed in all samples upon addition of NHS, which is arguably ascribable to the action of unknown components of human serum. Apyrase, which hydrolyses ATP to AMP and inorganic phosphate, strongly reduces FS3+NHS-induced [Ca^2+^] spikes in SCs in co-cultures (Fig. 2C and Movies 2 and 4).

### Neuronal ATP triggers cAMP production in Schwann cells

**Fig. 3.**
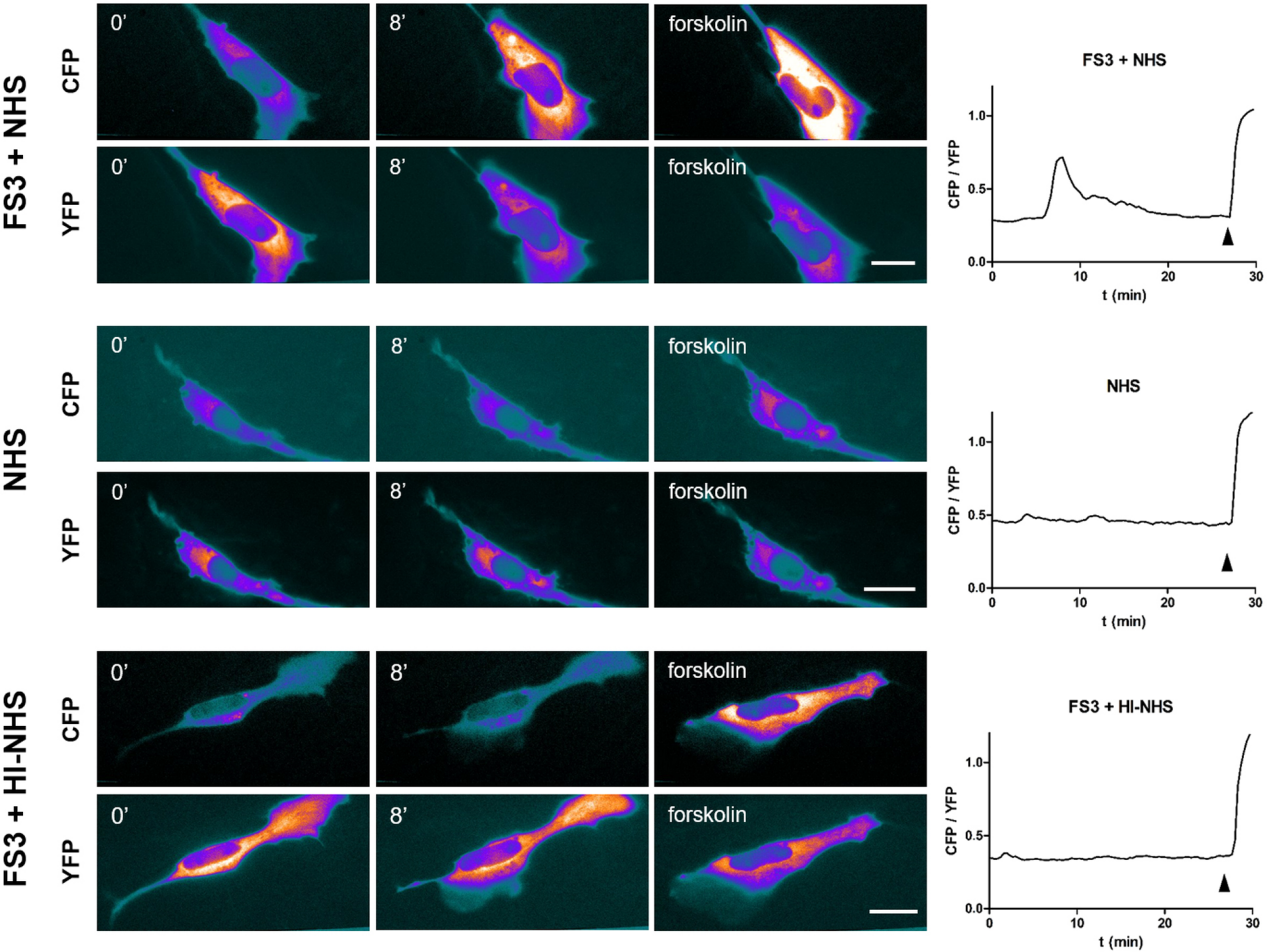
**Neuronal ATP triggers cAMP production in Schwann cells co-cultured with neurons.** SCs in co-cultures with SCMNs were transfected with the EPAC H187 sensor and FRET measured during exposure to FS3+NHS (complex added at *t*=1 min), NHS or FS3+HI-NHS. Forskolin (25 µM) was added at the end of the experiment (*t*=28 min, arrowhead) as a positive control. FRET causes a decrease in YFP fluorescence and a parallel increase in CFP intensity, as indicated by the pseudocolor images (blue, low fluorescence; white, high fluorescence). One representative experiment out of at least three for each condition is shown. Scale bars: 10 µm. Quantification is shown in the right panels, where FRET (CFP/YFP) is expressed as the ratio between the donor (CFP) and the acceptor (YFP) signals (corrected for the background).

Among the intracellular pathways through which purinergic receptors transduce the extracellular ATP input (Fields and Burnstock, 2006), we investigated the possible involvement of the second messenger cyclic AMP (cAMP), whose production is elicited by activation of type P2Y purinergic receptors. Primary SCs respond to exogenous ATP by raising their cAMP content (Negro et al., 2016). Accordingly, cAMP levels were imaged in SC and neuron co-cultures transfected with a new-generation EPAC (exchange protein directly activated by cAMP) probe (Klarenbeek et al., 2015) before and after exposure to FS3+NHS (Fig. 3; Fig. S1). Within a few minutes of FS3+NHS addition we observed a cAMP rise in SCs, with a peak at ∼8-10 min. Forskolin was added at the end of the experiment to reach maximum signal. No changes in cAMP levels were detected with NHS or FS3+HI-NHS (Fig. 3).

### Neuronal ATP induces CREB phosphorylation in Schwann cells

**Fig. 4.**
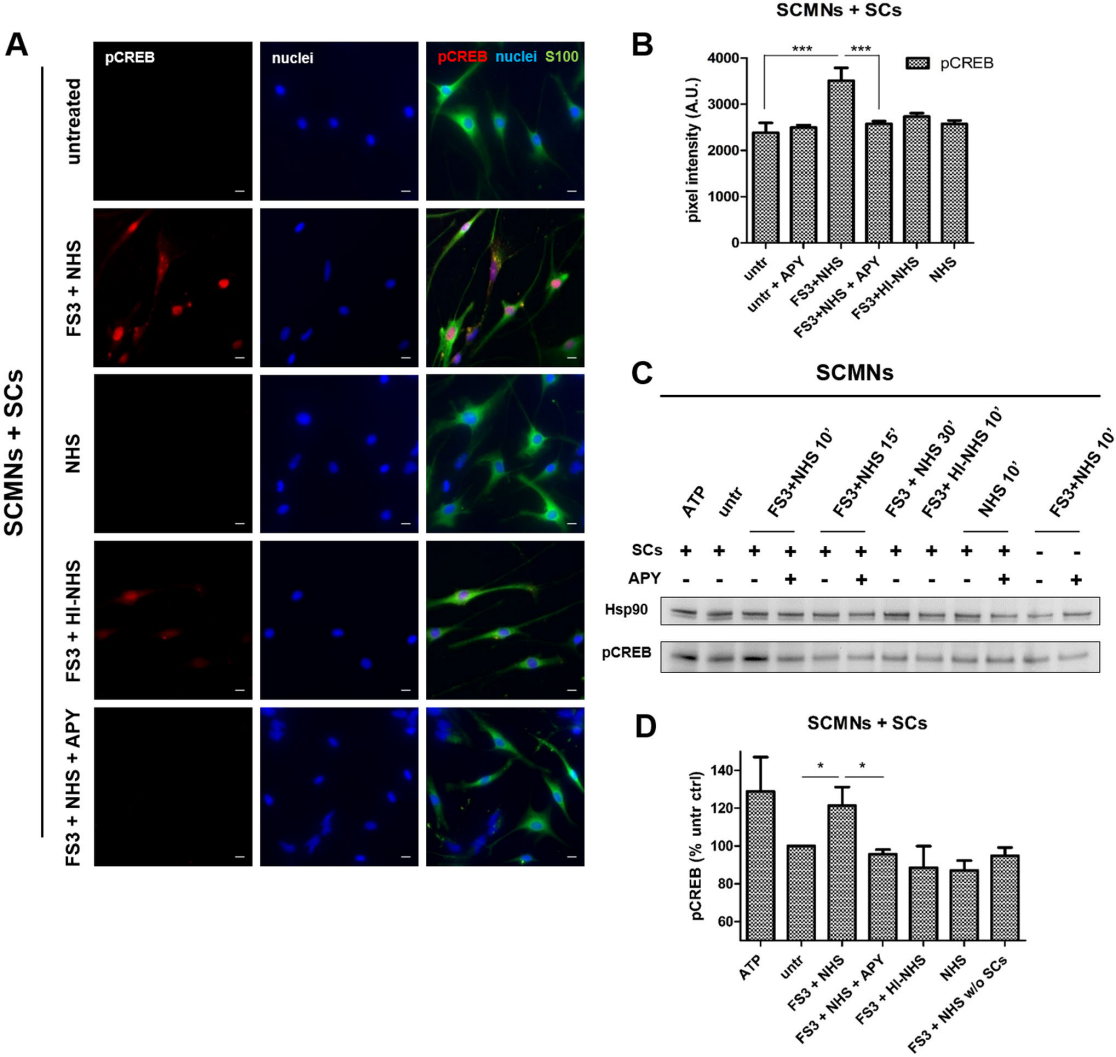
**Neuronal ATP induces CREB phosphorylation in Schwann cells in co-culture with neurons exposed to anti-GQ1b antibody plus complement.** (A) Immunofluorescence of SCMN and SC co-cultures exposed for 10 min to saline, FS3+NHS, NHS, FS3+HI-NHS or FS3+NHS plus apyrase. SCs are S100-positive cells (green), phospho-CREB is revealed by a specific antibody (red), nuclei are stained by Hoechst (blue). Scale bars: 10 µm. (B) Quantification of phospho-CREB in S100-positive cells. ***P<0.001; *N*=5. (C) Representative western blot showing phospho-CREB levels in SCMN and SC co-cultures exposed to FS3+NHS for 10, 15 and 30 min (as indicated), and their reduction by apyrase. At 10 min incubation, phospho-CREB signal is significantly higher in FS3+NHS samples with respect to untreated co-cultures. ATP 100 µM is used here as positive control. No phospho-CREB is detectable in neurons exposed to FS3+NHS, demonstrating that phospho-CREB signal in co-culture lysates derives from SCs. (D) Quantification of western blot in C. Data are normalized to the housekeeping protein Hsp90 and expressed as percentage of the untreated control. *P<0.05; *N*=5 (Student's *t*-test, unpaired, two-tailed).

The extracellular regulated kinases ERK1/2 and Jun regulated kinase (JNK) are crucial for the SC plasticity required for peripheral nerve regeneration (Napoli et al., 2012; Arthur-Farraj et al., 2012). The ERK1/2 pathway is also activated in SCs co-cultured with neurons upon exposure to FS3+NHS (Rodella et al., 2016). The transcriptional factor CREB, which is known to participate in many cellular processes and in neuron-glia communication, is among the targets of both ERK1/2 and cAMP pathways (Tabernero et al., 1998). CREB is phosphorylated in isolated SCs treated with ATP (Negro et al., 2016). Immunofluorescence on FS3+NHS-treated co-cultures revealed an increase in phospho-CREB signal in SC nuclei (Fig. 4A,B); this observation was further confirmed by western blot results (Fig. 4C,D). CREB activation depends on ATP, as apyrase strongly reduces phospho-CREB levels.

## DISCUSSION

The major finding reported here is an early release of ATP by neurons in the present *in vitro* model of MFS. ATP contributes to eliciting an increase in cytosolic [Ca^2+^] in the form of spikes and in cAMP content in co-cultured SCs, with an ensuing phosphorylation of the transcription factor CREB: these pathways play an important role in SC pro-regenerative behavior. The present results contribute to define the molecular mechanisms at the basis of the pathogenesis and reversibility of MFS, and emphasize the role of SCs in the regenerative process associated with the disease.

### A defined *in vitro* cellular model of MFS

MFS is caused by the complement-mediated autoimmune attack of peripheral nerves: MAC deposition takes place at neuronal surface both *in vitro* and *in vivo*, with ensuing intracellular calcium overload, loss of mitochondrial functionality, cytoskeletal alterations and nerve terminal paralysis (Plomp and Willison, 2009; Rodella et al., 2016). We have recently shown that the crucial pathogenic steps of MFS can be reproduced *in vitro* by exposing primary neurons to the combination of the monoclonal anti-GQ1b/GT1a IgG antibody (FS3) plus NHS as a source of complement (Rodella et al., 2016). FS3 was obtained by inoculating mice with a lysate of heat-killed *Campylobacter jejuni*, the infectious agent most frequently associated with MFS (Koga et al., 2005). Because more than 90% of MFS cases display IgG autoantibodies against GQ1b (Chiba et al., 1993), the FS3+NHS complex may be well considered as the ‘pathogen’ that reproduces the disease *in vitro* and *in vivo* (Rodella et al., 2016). Within a few minutes of the addition of the complex, varicosities appear along neurites: these are sites of massive cytosolic [Ca^2+^] increase causing rounding and dysfunction of mitochondria, which produce H_2_O_2_. This reactive oxygen species rapidly diffuses across membranes and reaches co-cultured SCs, triggering their pro-regenerative responses (Rodella et al., 2016). It is likely that there are additional neuronal signaling molecules with a role in the reversibility of this peripheral degenerative neuropathy.

### ATP: an alarm molecule involved in neuron-Schwann cell cross-talk and in Schwann cell activation

ATP is best known as an energy-rich molecule involved in many biochemical pathways, but it is also widely recognized as an extracellular danger-signaling molecule. ATP was found to be co-released along with acetylcholine at NMJs more than 40 years ago (Silinsky, 1975), and it interacts with both neurons and SCs. ATP can be sensed by SCs, which express an array of purinergic receptors on their surface. ATP signaling through purinergic receptors can engage several important intracellular pathways such as calcium, cAMP, inositol-1,4,5-trisphosphate, phospholipase C and several others (Fields and Burnstock, 2006).

Here, we show that ATP is a signaling molecule involved in the cross-talk between degenerating neurons and SCs in a cellular model of MFS. Neuronal ATP triggers cytosolic [Ca^2+^] spikes, cAMP increase and CREB phosphorylation in SCs in co-cultures. We recently reported that intracellular [Ca^2+^] rises in neurons exposed to FS3+NHS (Rodella et al., 2016). When neurons are co-cultured with SCs and treated with the anti-ganglioside antibody plus complement, cytosolic calcium levels increase in neurites, and this is temporally followed by cytosolic [Ca^2+^] spikes in SCs. This effect is, at least in part, ATP dependent, as it is reduced by pretreatment with apyrase. Apyrase failed to completely abolish calcium signaling in SCs, as expected on the basis of the fact that several mediators derived from degenerating neurons contribute to the process.

Upon cut or crush of the sciatic nerve, SCs undergo a profound reprogramming to sustain recovery of nerve function. They contribute to myelin clearance by phagocytosis, upregulate neurotrophic factors and surface proteins that promote axonal elongation; they also increase the survival of injured neurons, and recruit immune cells (Jessen and Mirsky, 2016). At least part of the transdifferentiation of SCs toward a regenerative phenotype relies on cAMP signaling. This process is less well defined for PSCs, which are different to myelinating SCs. As phagocytosis relies on cAMP (Pryzwansky et al., 1998), it is likely that this pathway could also be important for PSCs that actively participate in the clearance of nerve debris by phagocytosis (Son et al., 1996; Duregotti et al., 2015). Indeed, cAMP is produced by SCs co-cultured with injured neurons.

Following a variety of stimuli, protein kinase A, activated by cAMP, engages the transcription factor CREB, which recognizes the cAMP-responsive element in target genes, initiating their transcription (Shaywitz and Greenberg, 1999). CREB phosphorylation takes place in SCs during nerve development and after nerve transection (Stewart, 1995). Accordingly, ATP-dependent CREB phosphorylation occurs in SCs co-cultured with injured neurons.

Although these results were obtained using a monoclonal antibody specific for GQ1b, because it is the pathogen responsible for MFS, they are likely to be valid for all complement-binding antibodies specific for other polysialogangliosides of the presynaptic membrane.

### Conclusions

We have shown here that ATP acts as an alarm messenger and is rapidly released by neurons exposed to anti-GQ1b/GT1a antibody plus complement – the ‘pathogen’ responsible for the autoimmune attack of peripheral nerves in MFS. ATP activates calcium, cAMP and phospho-CREB signaling pathways in SCs, which are expected to drive the complex response of these cells to impairment of nerve function.

## MATERIALS AND METHODS

### Chemicals and primary cell culture

The mouse monoclonal antibody (FS3, isotype IgG2b-κ) was previously characterized for its specificity towards gangliosides GQ1b and GT1a (Koga et al., 2005). Normal human serum (NHS) from a pool of human healthy males AB plasma (Sigma-Aldrich, H4522, lot SLBG2952V) was employed as a source of complement. Rat primary culture of SCMNs, CGNs, primary SCs and their co-culture were described previously (Rigoni et al., 2004, 2008; Duregotti et al., 2015). Unless otherwise stated, all reagents were purchased from Sigma.

All experimental procedures involving animals and their care were carried out in accordance with National laws and policies and with the guidelines established by the European Community Council Directive (2010/63/UE) and were approved by the local authority veterinary services.

### ATP measurement

ATP was quantified in the supernatants of primary neurons using the commercial ATP Lite One-Step kit (Perkin-Elmer), which relies on a luminescence-based ATP-dependent reaction. Cells were exposed for different time periods to FS3 (0.1 µg/ml) in combination with NHS (0.5% v/v), as well as to appropriate control conditions. Ecto-ATPase and NHS-ATPase activity was quenched by the addition of 1 mM sodium orthovanadate in the incubation medium. For each well, the ATP released was estimated as the ratio between ATP in the supernatant and total cellular ATP (cells were lysed using Triton X-100, 0.5% v/v) and expressed as a percentage of the untreated control. A brief centrifugation of supernatants was performed to eliminate cell debris before measurements. Luminescence was measured with a luminometer (Infinite M200 PRO, Tecan).

### Lactate dehydrogenase activity assay

CGNs plated on 24-well plates (250,000 cells/well) were exposed to saline, NHS, FS3 (0.1 µg/ml) and NHS (0.5% v/v), or FS3+HI-NHS for 30 min. Supernatants were collected and lactate dehydrogenase (LDH) activity was measured following the manufacturer's instructions (Sigma) and normalized by total cellular LDH activity from cell lysates. Each sample was expressed as percentage of the positive control (cells were lysed using Triton X-100, 0.5% v/v).

### Calcium imaging

Co-cultures of primary neurons and SCs were loaded for 10 min with the calcium indicator Fluo-4AM (4 μM) (Invitrogen). Cells were then washed and moved to the stage of an inverted fluorescence microscope (Eclipse-Ti; Nikon Instruments) equipped with a perfect focus system (PFS; Nikon Instruments) and high numerical aperture oil immersion objective (60×). Calcium signals were recorded in control samples, in samples exposed to 0.1 µg/ml FS3*+*0.5% NHS (v/v), NHS alone, or FS3+HI-NHS, with excitation of the fluorophore performed at 465-495 nm by means of an Hg arc lamp (100 W; Nikon). Emitted fluorescence was collected at 515-555 nm and measured in a selected region of interest containing cell cytosol corrected for background fluorescence. Images were acquired for 30 min every 20 s. In some experiments, 1.5 U/ml apyrase was added 15 min before image acquisition and left throughout.

### cAMP detection

EPAC-S^H187^, a fourth generation EPAC-based FRET probe for cAMP detection was employed. This sensor consists of the cAMP-binding protein EPAC sandwiched between mTurquoise2, a very bright and bleaching resistant donor fluorescent protein, and a novel acceptor cassette consisting of a tandem of two Venus fluorophores (Klarenbeek et al., 2015). Briefly, SCs co-cultured with neurons were transfected with 1 μg of the probe with Lipofectamine 2000 (Life Technologies). Experiments were performed 24 h after transfection. Cells were monitored using an inverted fluorescence microscope (Eclipse-Ti; Nikon Instruments) equipped with the perfect focus system (PFS; Nikon Instruments). Excitation of the fluorophore was performed by an Hg arc lamp (100 W; Nikon) using a 435 nm filter (10 nm bandwidth). YFP and CFP intensities were recorded with a cooled CCD camera (C9100-13; Hamamatsu) equipped with a 515 nm dichroic mirror at 530 nm (25 nm bandwidth) and 470 nm (20 nm bandwidth), respectively. Signals were digitized and FRET was expressed as the ratio between donor (CFP) and acceptor (YFP) signals (CFP/YFP) corrected for background. Co-cultures were pre-incubated with 1 mM sodium orthovanadate as a general ATPase inhibitor for 5 min, then, after 1 min recording, cells were exposed to 0.1 µg/ml FS3+0.5% v/v NHS, NHS alone, or FS3+HI-NHS. A final stimulation with 25 μM forskolin was performed at the end of each experiment to maximally raise cAMP levels.

### Western blotting

Following treatment, samples were lysed in lysis buffer [4% SDS, 0.125 M Tris-HCl, pH 6.8, protease inhibitor cocktail (Roche) and phosphatase inhibitor cocktail (Sigma)]. Total lysates from SCMN and SC co-cultures (treated as above) were loaded on precast 4-12% SDS-polyacrylamide gels (Life Technologies) and transferred onto nitrocellulose paper in a refrigerated chamber. After saturation, membranes were incubated overnight with a rabbit polyclonal antibody against phospho-CREB (p-CREB) (1:1000, Cell Signaling, 9198S), followed by a secondary anti-rabbit secondary HRP-conjugated antibody (Calbiochem, 1:2000, 401393). Chemiluminescence was developed with the Luminata TM Crescendo (Millipore) or ECL Advance western blotting detection system (GE Healthcare), and emission measured with ChemiDoc XRS (Bio-Rad). For densitometric quantification, the bands of interest were normalized to the housekeeping protein Hsp90 (mouse monoclonal, 1:1000, BD Transduction Laboratories, 610419). Band intensities were quantified on the original files with the software Quantity One (Bio-Rad). None of the bands reached signal saturation. In some samples, apyrase (1.5 U/ml) was added 15 min before experimental treatments and left throughout.

### Immunofluorescence

After treatment, neuron-SC co-cultures (treated as above) were fixed for 10 min in 4% (w/v) paraformaldehyde (PFA) in PBS, quenched [0.38% (w/v) glycine, 50 mM NH_4_Cl in PBS], and permeabilized with 0.3% Triton X-100 in PBS for 5 min at room temperature (RT). After saturation with 3% (v/v) goat serum in PBS for 1 h, samples were incubated with primary antibodies (anti-phospho-CREB, 1:500, Cell Signaling, 9198S; anti-S100, 1:200, Sigma, S2532) diluted in 3% (v/v) goat serum in PBS overnight at 4°C, washed, and then incubated with Alexa-conjugated secondary antibodies (1:200, Life Technologies) for 1 h at RT. Coverslips were mounted ProLong Diamond (Thermo Fisher Scientific) and examined by epifluorescence (Leica CTR6000) microscopy.

### Statistical analysis

The sample size (*n*) of each experimental group is described in each corresponding figure legend; at least three biological replicates were performed. GraphPad Prism software was used for all statistical analyses. Quantitative data displayed as histograms are expressed as means±s.e.m. (represented as error bars). Results from each group were averaged and used to calculate descriptive statistics. Significance was calculated by Student's *t*-test (unpaired, two-side). *P*-values less than 0.05 were considered significant.
